# Editorial: Insights in Aging and Public Health: 2021

**DOI:** 10.3389/fpubh.2022.929418

**Published:** 2022-06-10

**Authors:** Marcia G. Ory, Matthew Lee Smith

**Affiliations:** ^1^Department of Environmental and Occupational Health, School of Public Health, Texas A&M University, College Station, TX, United States; ^2^Center for Population Health and Aging, Texas A&M University, College Station, TX, United States

**Keywords:** aging and public health, insights, population aging, resilience, social connectedness, intergenerational health, lifestyle factors, built environment

## Introduction

Our global society is changing. Now in the third decade of the twenty-first century, the achievements made by scientists have led to major advancements in the fast-growing field of Aging and Public Health. As indicated by the United Nations Declaration of the Decade of Healthy Aging (2021–2030), there is global interest in understanding determinants of healthy aging and strategies to improve the lives of older people, their families, and the communities in which they live (1, 2). As such, the field of public health and aging must constantly evolve and adapt alongside the ongoing changes in population growth and demographics, social and physical environments, and policy and other drivers of health-related costs (3). Further, the indicators of risk and markers of success have assumed new meaning as new societal needs/challenges have emerged and our initiatives have targeted new populations and health behaviors (4).

This inaugural “*Insights in Aging and Public Health: 2021*” Research Topic includes forward-looking contributions focused on new insights, novel developments, current challenges, latest discoveries, recent advances, and future perspectives in the field of Aging and Public Health. Taking an inclusive view of different article types for advancing knowledge and practice, this Research Topic includes original research, brief research reports, systematic and mini-reviews, perspective and opinion pieces, and community case studies. It also reflects the geographic diversity of aging and public health researchers with contributors from Asia, Europe, the Middle East, Oceania, and the Americas. We briefly overview the selected articles, which we have organized around four main themes: (1) new ways of looking at aging; (2) resilience and social connectedness/isolation; (3) the power of intergenerational relationships; and (4) physical activity and built environment.

## Themes

### Theme 1

The Research Topic begins with new ways of looking at aging and its implications for clinical practice. The heterogeneity of the older population is a classic aging principle (5). In their insightful perspective, Jaul and Barron propose a four-stage system for characterizing aging in terms of chronological age, functional status/disease burden, and life expectancy. The value of this system is its use in clinical decision-making to improve clinical care, provide personalized anticipatory guidance, and better understand the impact of functional status on the quality of life of geriatric patients. A related review by Adja et al. emphasizes the importance of taking a patient-centered, community-based approach to preventing and managing frailty. Specifically, they call for a stronger alliance between public health and primary care to view frailty as a multidimensional construct and understand clinical perspectives within the broader societal context.

### Theme 2

Resilience and social connectedness have become major themes in understanding how older adults have managed throughout the COVID-19 pandemic (6–9). Even before the pandemic began, Madsen et al. foreshadowed the value of resilience for individuals and communities in the face of disaster. Their contribution is reframing the dialogue from a deficit view of aging to one that recognizes personal-level capacities that, in turn, promote community resilience. Their review provides valuable insights into everyday strategies to increase personal capacities, collective capacities, and/or community resources that can help buffer the range of adversities older adults encountered during the COVID-19 pandemic and are likely to encounter into the future.

There is a growing literature on the association of social isolation with a myriad of poor health outcomes, especially among the older population (10–12). Lynch et al. examined rates of social isolation by geographic location among a cohort of older drivers across the United States. Their brief research report revealed higher rates of social isolation in non-urban areas, although not significantly different. While this study was limited in the diversity of its sample, a major contribution was the use of a standard social isolation measure for improved measurement precision and recommendation for measurement standardization in future research.

### Theme 3

Attention to social connectedness has led to a renewed interest in intergenerational research (13). In their opinion piece, Wong et al. make the argument that the involvement of younger people is essential to achieving healthy aging for all. They advocate for innovative intergenerational programs, education about aging to disrupt aging stereotypes, and participatory involvement of youth. This new vision will help create a new social contract between young and old to better address the Decade of Healthy aging's four action areas to change perceptions of age and aging, foster age-friendly environments, deliver age-responsive care, and provide quality long-term care (1, 2). A related analytical narrative review by Vaccaro et al. focuses on intergenerational communication and/or intergenerational social support to improve health, disease management, and/or participation in health research. Of special interest is the understanding about how such intergenerational models can better engage low-income minority populations in their healthcare. This study proposed a model to improve family intergenerational communication among Black and Hispanic older adults through additional qualitative research that addresses enablers and barriers to improved medical care, health behaviors, and health outcomes.

Novel approaches are needed to support intergenerational programs during the COVID-19 pandemic. In their case study, Kennedy-Behr et al. described the creation of an impactful intergenerational program model, its adaptation to community needs, and the employment of new online delivery models. A key success was co-designing the program with key stakeholder involvement, which included aged care providers, university staff and students, childcare staff, and aged care residents. The findings around their five themes (i.e., connection, skilled facilitators, exploration of past and present roles, a wish for continuity, and online challenges) provide evidence about the success of intergenerational programming, even with a pivot to virtual delivery.

### Theme 4

Lifestyle factors and the built environment have been key factors associated with healthy aging, especially physical activity (14, 15). Physical inactivity has been associated with a myriad of poor health and functional outcomes, especially among older adults who are characteristically less physically active than other age groups. González-Ravé et al. examined the effects of a 10-week multicomponent exercise program among older women. The major strength of this original research was delineating different exercise protocols and comparing them on standardized functional and clinical outcomes. A unique aspect was its detraining period, enabling an assessment of intervention sustainability. The training program's confirmed benefits on some strength- and clinically-related outcomes is consistent with prior research about the value of physical activity regimens, even among older adults. However, due to sample sizes, it was not possible to assess the relative value of the different training protocols. Hence, further research is recommended to identify the relative advantage of different training programs on various functional and clinical outcomes over time.

Fogaça et al. conducted a systematic review to better understand the benefits of traditional Chinese mind-body therapies, which combine physical movements with relaxation and breathing techniques. This study resulted in an evidence map that provides an at-a-glance visualization of the positive effects of combining mind-body approaches (e.g., Tai Chi, Qigong, Yoga). Evidence maps are important for advancing current knowledge and practice about complementary therapies and can offer new options for older adults around the world to engage in health-promoting lifestyles.

While walking is known to promote health and functioning in old age, the built environment can act as a facilitator or barrier to walking frequency (16–18). Wu et al. conducted original research to identify the relationship between the built environment and walking and whether threshold characteristics of the built environment were associated with different walking levels. This research revealed non-linear relationships and concluded that the relationship is more complex than previously acknowledged. For example, low and medium population density may support walking frequency, but high population density may have only a small effect on utilitarian walking. Such in-depth analyses of different built environment characteristics can inform community/city planners and policymakers about strategies to support active-aging environments.

## Conclusion

This Research Topic of 10 articles broadly encompasses a variety of health-related issues across the socioecological spectrum that impact older adults and the communities in which they live. As such, this Research Topic highlights the progress made in the past decade as well as existing and future challenges still needing to be addressed. Findings from this Research Topics are intended to inspire, inform, and provide direction to researchers and practitioners in the field of aging and public health.

## Author Contributions

Both authors listed have made a substantial, direct, and intellectual contribution to the work and approved it for publication.

## Conflict of Interest

The authors declare that the research was conducted in the absence of any commercial or financial relationships that could be construed as a potential conflict of interest.

## Publisher's Note

All claims expressed in this article are solely those of the authors and do not necessarily represent those of their affiliated organizations, or those of the publisher, the editors and the reviewers. Any product that may be evaluated in this article, or claim that may be made by its manufacturer, is not guaranteed or endorsed by the publisher.
